# From domination to partnership: Lab-trained microorganisms for environmental bioremediation

**DOI:** 10.1038/s44319-025-00681-5

**Published:** 2026-01-03

**Authors:** Victor de Lorenzo

**Affiliations:** National Center of Biotechnology CSIC, Systems Biology Department, Campus de Cantoblanco, Madrid, 28049 Spain

**Keywords:** Biotechnology & Synthetic Biology, Economics, Law & Politics, Evolution & Ecology

## Abstract

Despite advances in synthetic biology and ecological understanding, the deployment of engineered microorganisms for bioremediation remains stalled due to outdated containment-centric narratives and regulatory frameworks. A shift from a logic of *control* to one of *care and stewardship*, is needed for a more responsible, culturally adjusted and scientifically grounded path toward leveraging biotechnology for planetary repair.

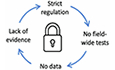

The release of genetically engineered microorganisms into the environment has remained one of the most controversial and least explored frontiers in biotechnology. More than four decades after the early debates on the risks of recombinant DNA technology, field deployment of engineered bacteria to eliminate toxic waste and industrial and urban emissions remains practically frozen, particularly in Europe (Chemla et al, 2025).

But the planet now faces different challenges than those that dominated the conversation when concerns and opportunities of the then incipient recombinant DNA technology were first discussed in the 1970s. On the one hand, we have the ever-increasing problems of climate change, plastic pollution, persistent and ubiquitous chemicals in the environment, and growing desertification (Richardson et al, 2023). On the other hand, the profound advances in microbial ecology, systems biology, and synthetic biology have transformed our understanding of biological systems and have produced an amazing arsenal of molecular tools for their manipulation.

Yet, leveraging the possibilities of advanced biotechnology for the sake of a better environment still faces a major bottleneck: the delivery of human-designed agents to the intended target sites (de Lorenzo, 2022). It is one thing to engineer a strain with an environmentally useful trait—whether enhanced CO₂ fixation, xenobiotic breakdown, or explosives detection—and grow it in a Petri dish or a small bioreactor, and quite another to deliver that function effectively and safely at a scale required to address environmental problems. On these bases, the time has come for a new narrative: one grounded in ecological realism, evolutionary logic, and the recognition that engineered microorganisms are not alien intrusions but laboratory-trained extensions of life’s creativity. Rather than policing an unachievable ideal of absolute containment and control, governance should embrace traceability, accountability, and long-term stewardship. This shift from ‘control to care’ (Szymanski et al, 2024) would consider engineered microbes as environmental probiotics capable of repairing degraded ecosystems.

“… engineered microorganisms are not alien intrusions but laboratory-trained extensions of life’s creativity”

## Who is afraid of genetic programming of bacteria for environmental benefit?

**Figure 1:**
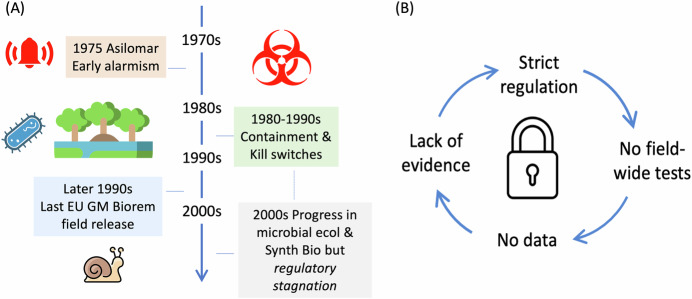
From caution to stagnation. (**A**) The sketch illustrates how early fears, outdated ordinance, and lack of field data have created over the years a regulatory deadlock cycle, as represented in (**B**). Ruling frameworks have remained largely anchored in the 1970s mindset despite scientific, ecological, and technological breakthroughs in the meantime.

Environmental biotechnologists have long envisioned using engineered microbes to address contamination and degradation of soil, freshwater, marine niches, and even air. As early as the 1980s, scientists proposed using genetically modified bacteria to degrade hydrocarbons and xenobiotic compounds, detoxify heavy metals or improving water quality (Wilson and Lindow, 1993). These ambitions coincided with the onset of recombinant DNA technology, while both ecological knowledge and genetic tools available for reprogramming the metabolism of microbes were rudimentary. Over the ensuing years, the absence of robust large-scale ecological models, combined with the dearth of real-world case studies and public concerns—some altogether legit, others imaginary—about the safety of agents created in the laboratory, resulted in a regulatory environment governed more by fear than evidence. In Europe, the result has been decades of paralysis (Fig. 1).

Since the late 1990s, not a single field release of a genetically engineered bacterium for bioremediation has been approved. This regulatory stagnation has discouraged investment, stifled innovation, and prevented the generation of much-needed field-testing data. The dilemma is circular: no field trials mean no data, and no data mean no field trials. Meanwhile, the world has changed dramatically. Global warming accelerates soil degradation, water scarcity, and pollution. Plastic contamination, microplastics, persistent organic pollutants, and polyfluorinated chemicals accumulate across ecosystems. But it also happens that contemporary systems and synthetic biology provide conceptual and molecular assets for programming biological functions with precision and predictability that were unimaginable in the 1980s (Jones et al, 2024). Yet environmental biotechnology involving engineered microorganisms remains constrained by a regulatory architecture frozen in time.

“… environmental biotechnology involving engineered microorganisms remains constrained by a regulatory architecture frozen in time.”

The public perception of genetic engineering is inseparable from the early concerns surrounding recombinant DNA. The 1975 Asilomar Conference—often celebrated as an ethical milestone—had also unintended consequences (Watson et al, 1982). Its cautious framing helped establish the notion that engineered life forms were categorically distinct and inherently hazardous. Scientists themselves, in raising alarms, amplified public concern (Danchin, 2010). The *arsonist firefighter* phenomenon—warning society of dangers one has oneself introduced—played a significant role in shaping early perceptions.

The vocabulary that emerged from this period reinforced the sense of danger: containment, kill switches, suicide circuits, firewalls (Ramos et al, 1995). These terms implied that engineered microbes must be controlled, neutralized, or kept behind metaphorical barricades. This linguistic framing has had lasting consequences, as it displaced discussion of benefits and opportunities and put biotechnology on a defensive footing from which it has never fully escaped. Early missteps in communication and the perception of profit-driven biotech companies prioritizing commercial gain over public safety deepened societal skepticism, if not outright rejection, of releasing modified bacteria into the environment. When industry appeared motivated primarily by economics, and scientists highlighted risks, public sympathy for the field naturally waned, replaced instead by fear and apprehension.

It is revealing that the prevailing scientific response to these concerns—still evident in contemporary synthetic biology—has been to describe genetically designed organisms as controllable machines (Andrianantoandro et al, 2006) rather than as added participants in natural ecosystems. From this hard-core engineering perspective, any risks posed by rationally generated biological entities are presumed to be solvable through additional layers of containment, guaranteeing function only within predetermined spatial and temporal boundaries and minimizing interactions with other forms of life (Pei et al, 2022; Ramos et al, 1995). In other words, the concept of *orthogonalization*—a foundational, almost talismanic concept in synthetic biology (Costello and Badran, 2021).

## Is containment the way to go?

Containment has been the cornerstone of biosafety for engineered microorganisms, specifically for those to be deliberately released for bioremediation (Ramos et al, 1995; Urgun-Demirtas et al, 2006). Over decades, researchers have devised ingenious systems—auxotrophic dependencies, kill switches triggered by environmental cues, synthetic addictions, orthogonal transcriptional systems, genetic firewalls, and many more (Pei et al, 2022). Yet even the most sophisticated systems remain vulnerable to mutation, recombination, or ecological compensation. The best laboratory-tested containment circuit achieves escape frequencies around 10^−^¹¹—this is remarkable, but not absolute.

Ecological principles reveal why containment cannot guarantee confinement. Environmental microbial communities are dynamic, interconnected, and governed by competitive pressures and homeostatic mechanisms. Furthermore, they experience horizontal gene transfer among distant species (Soucy et al, 2015) and often mutate when subject to stress or through mere genetic drift. These forces cause introduced strains to be outcompeted or assimilated in the long run. In fact, the few historical large-scale field releases of engineered bacteria (Peters et al, 1997) showed rapid decline or assimilation, with no evidence of ecological disruption or runaway propagation.

The containment paradox therefore arises: it is conceptually reassuring but ecologically unrealistic. Endowing engineered bacteria with containment devices often creates the illusion of control while obscuring the inherent fluidity and adaptability of natural microbial ecosystems. The logic of control—so appealing to engineers and widely assumed to minimize the risks of biological agents—may ultimately be ill-suited to bacteria released into the environment. Consider aviation, perhaps the most iconic domain of successful engineering: its extraordinary safety record is largely the product of learning from accidents, each one carefully analyzed to prevent future failures. In contrast, bacterial genetic engineering has experienced virtually no comparable accidents from which to learn (Schmidt and de Lorenzo, 2012). Moreover, the stringent regulatory barriers that limit field trials further restrict the generation of empirical data. As a result, the precautionary confidence placed in existing containment systems remains, for the most part, hypothetical rather than evidence-based. Despite intense research efforts, currently available genetic safeguards are still considered insufficiently reliable—at least in the EU—for the environmental release of engineered microbes for bioremediation due to mutational escape and context-dependence. At the same time, several designs explicitly highlight their potential use as ‘passcodes’ or proprietary chassis to restrict unauthorized propagation or modification of strains, effectively repurposing biocontainment architectures as tools for intellectual-property protection and use limitation rather than environmental safety (Chan et al, 2016). In any case, if the logic of control fails as a safety feature of bacteria for extensive bioremediation, what can be done instead?

“The containment paradox therefore arises: it is conceptually reassuring but ecologically unrealistic.”

## From domination to accompaniment

In order to overcome this impasse and move on to making *biotechnology beyond containment* a reality, we advocate a necessary shift from the “logic of control to the logic of stewardship” (Szymanski et al, 2024). One step in this direction is the development of new concepts and molecular tools for traceability, that is, for ensuring that engineered microbes should not be controlled and dominated, but identified, monitored and, if necessary, recalled. This can be achieved, among others, through genomic barcodes (Tellechea-Luzardo et al, 2022) DNA watermarks (Gallegos et al, 2020), or sequence tags embedded into the genomes of strains (Berezin et al, 2024) engineered for release which act as unique identifiers. These can be linked to digital twins—virtual representations, behavior models, deployment records, and monitoring/clearance instructions (Tellechea-Luzardo et al, 2022).

Unlike control and containment, traceability promotes transparency, accountability, and adaptive governance. It is far more compatible with the realities of microbial ecology—how communities behave, how they integrate newcomers, and how genes and functions circulate within ecosystems. Instead of attempting to erect barriers against gene flow—an ultimately futile endeavor—traceability allows us to follow designed organisms destined for release throughout their entire bioremediation lifecycle. It is important to acknowledge, however, that any engineered DNA sequence will be subject to evolutionary pressures and may be erased, diluted, or assimilated over time, while beneficial functions can be transferred to the indigenous microbiota (Peters et al, 1997). This evolutionary dynamic reframes persistence: not as a failure or a risk in itself, but as a potential pathway for the integration and reinforcement of introduced functions within existing ecological networks.

## A matter of perspective

Much of the debate on releasing engineered bacteria for bioremediation gravitates around the notion of *natural* versus *artificial*and where to draw the boundary between them. But perceptions are far from universal: they are shaped by cultural traditions that strongly influence public attitudes toward biotechnology. In much of Europe and North America, nature is imagined as a pristine state that must be protected from human disturbance. This notion, rooted in Romanticism and reinforced by regulatory practice, draws a sharp line between natural ecosystems and engineered interventions, encouraging a defensive posture grounded in precaution and containment. By contrast, East Asians tend to view humans and nature as parts of a single, dynamic continuum. Here, the boundary between natural and artificial is more permeable, and technological interventions are often interpreted as efforts to harmonize or improve natural processes rather than to violate them. Various indigenous perspectives similarly reject rigid dichotomies, emphasizing relationships, reciprocity, and the integration of new agents—biological or otherwise—into existing ecological and cultural networks. Other cultural landscapes, such as Latin America and the Mediterranean, occupy a middle ground in which concerns focus less on the act of modifying life than on issues of trust, governance, and control—particularly the suspicion that large corporations may pursue their own interests at the expense of local communities. Finally, techno-optimist cultures tend to downplay the distinction altogether, viewing nature as something that can be improved, optimized, or redesigned. These varying cultural framings shape how societies evaluate engineered organisms in the environment: whether they are seen as threats, partners, tools, or unwelcome intruders. Recognizing this diversity is essential for developing governance approaches that are responsive, legitimate, and culturally grounded rather than universally imposed.

A useful starting point for regulatory reform would be to move beyond the unhelpful binary between *naturally occurring microorganisms* and *genetically modified microorganisms* that currently dominates EU legislation. Instead, engineered strains should be understood along a continuum of laboratory-trained microorganisms: life forms shaped by different degrees of human influence but still operating within the familiar rules of biochemistry and evolution. This continuum begins with wild isolates brought into the laboratory for study and that are reintroduced without modification. Next would be strains that acquire adaptive traits through stress-induced mutagenesis or spontaneous variation, followed by recombinant organisms produced through conventional genetic engineering. At the far end lie rationally redesigned organisms built from available genetic and biochemical activities but assembled into new, engineered architectures. Despite their varying degrees of deliberate intervention, all these forms still obey the canonical biochemistry of the Central Dogma of Molecular Biology and can therefore be regarded as bona fide natural, at least in the sense that they operate within the same chemical and evolutionary space as their unmodified counterparts (de Lorenzo, 2010).

Recognizing this continuum challenges the assumption that laboratory-trained strains inevitably pose exceptional risks. All microorganisms—whether engineered or “natural”—can mutate, exchange genes, colonize new niches, or disappear under selective pressures. Most genetic engineering today remains within the realm of biological innovation, understood as novel combinations of pre-existing functions and parts, rather than the creation of genuinely new-to-nature codes or chemistries. Regulatory frameworks built on categorical distinctions fail to reflect this biological reality. Systems based on unfamiliar molecular architectures—xeno-nucleic acids, non-canonical amino acids, altered genetic codes (Schmidt and de Lorenzo, 2012) or mirror-image life (Adamala et al, 2024)—represent clear departures from naturally occurring biology and should indeed be subject to strict regulatory scrutiny. In other words, a practical regulatory boundary between natural and artificial biology could be drawn not on genetic criteria but on biochemical foundations.

## Towards a new narrative

**Figure 2:**
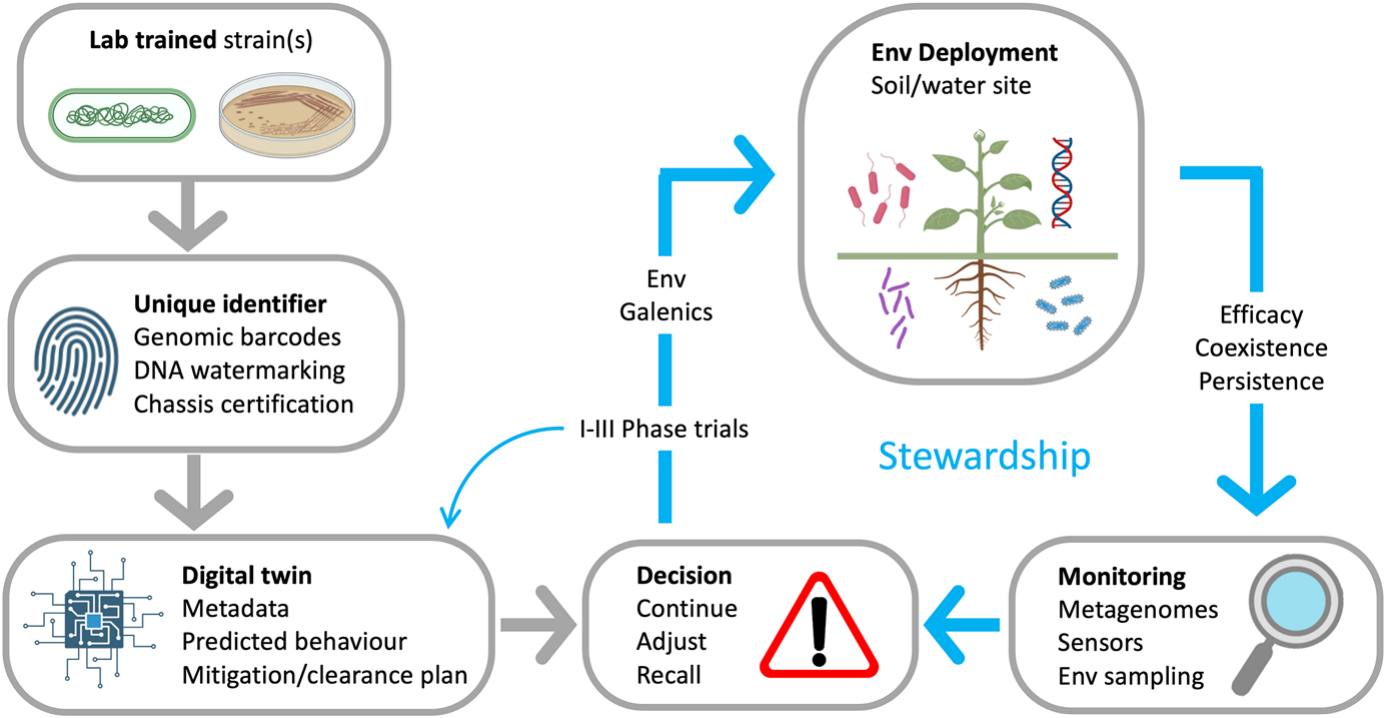
From containment to care: towards a governance model based on traceability and stewardship. The flow diagram links laboratory organisms to their environmental lifecycle and monitoring tools with an emphasis not on containment but on traceability, accountability, and continuous interaction with the environment. The roadmap begins with a laboratory-trained strain equipped with one or more environmentally relevant traits. Each strain receives a unique identifier linked to a digital twin that consolidates all available information about it. Depending on the depth of knowledge and supporting data, the strain can then advance through a series of phased trials modeled on the procedures used in the healthcare sector to approve new drugs. Insights from each phase are incorporated back into the digital twin. Once phase III is successfully completed, the strain may be deployed more broadly to the target site. After release, its performance, persistence, and any unintended effects on the resident microbiota could be monitored through metagenomic sampling and/or in situ or remote sensors. Based on the results, the release can either be scaled up further or recalled. This governance framework moves beyond traditional notions of containment and instead uses the microbial and physicochemical ecosystem itself as the scaffold for introducing beneficial activities.

Moving from ‘control to stewardship’ advocated in this Opinion requires rethinking biotechnology not as a means of dominating nature but as a form of partnership. Language plays a central role. Instead of speaking of kill switches, we might describe ecological anchoring mechanisms or spatiotemporal addiction circuits that tie designed microbes to their intended niches through positive dependencies rather than punitive failures. Similarly, instead of GMO releases for bioremediation, we could name these interventions as the deployment of environmental probiotics—microbial agents that restore ecological balance (Fig. 2). This shift of vocabulary is not just a marketing strategy: it reflects the deeper conceptual change of seeing engineered life as part of a mutualistic dialog with ecosystems, not an adversarial intrusion. Evolutionary “conversation” between designed systems and their environments may yield solutions that rational engineering alone cannot anticipate. Care involves ongoing monitoring, iterative learning, and long-term responsibility—an ethos that fits traceability-based governance.

“…instead of GMO releases for bioremediation, we could name these interventions as the deployment of environmental probiotics—microbial agents that restore ecological balance.”

This perspective contrasts with the dominant Western regulatory paradigm, which tends to frame engineered microbes primarily as hazards requiring stringent containment. Emphasizing relationships, reciprocity, and ecological integration can provide novel conceptual tools to reframe engineered organisms not as foreign intruders but as potential contributors to ecosystem repair and health. These notions can contribute to the necessary shift in narrative—from a focus on risk and control to one of repair, care and responsible partnership with living systems. And in turn, such reframing can ease acceptance of engineered microorganisms. Emerging indigenous biotechnology initiatives (Astolfi et al, 2025; Villanueva-Flores and Garcia-Atutxa, 2025) demonstrate how traditional ecological insights can complement modern molecular tools, as engineered microbes may not be seen as artificial threats but extensions of nature’s evolutionary processes. With responsible governance, transparent monitoring, and a renewed conceptual framework, they can become vital instruments of planetary repair. To harness this potential, biotechnology must—as advocated in this opinion—move beyond domination and containment toward an attitude of partnership with the living world.

“…biotechnology must […] move beyond domination and containment toward an attitude of partnership with the living world.”

In conclusion, the deliberate release of engineered microorganisms can be key for addressing the environmental challenges of the Anthropocene (Richardson et al, 2023). Yet regulatory and conceptual barriers rooted in outdated narratives focused on containment have prevented progress. A shift from control to care—anchored in traceability, stewardship, and ecological realism—is essential for unlocking the potential of laboratory-trained life forms.

## Supplementary information

Peer Review File
