# Supplementary material for: From domination to partnership: Lab-trained microorganisms for environmental bioremediation
Source: EMBO Rep. 2026 Jan 3;27(3):561–5. doi: 10.1038/s44319-025-00681-5 (PMC12895013; doi:10.1038/s44319-025-00681-5)
Supplement: Supplementary file 1 — Peer Review File [file 44319_2025_681_MOESM1_ESM.pdf]

EMBO reports

From domination to partnership: Lab-trained microorganisms for environmental bioremediation

Victor de Lorenzo

Corresponding author(s): Victor de Lorenzo (vdlorenzo@cnb.csic.es)

Review Timeline:

Submission Date:	11th Dec 25
Accepted:	15th Dec 25

Editor: Holger Breithaupt

Transaction Report: This article was editorially reviewed.
